# Quarterly Summary

**Published:** 1855-07

**Authors:** 


### QUARTERLY SUMMARY.

### DENTAL SCIENCE.

1.—*Sponge Gold.*—Dr. Arthur, in an article upon this subject in the News Letter, for October, points out the impossibility of using gold foil, except in the most simple cases, and in these even time and great labor are necessary to success. The doctor assumes that the objections to gold foil are obvious, while the only substances hitherto used, combining the good qualities of the foil with the proper amount of plasticity are the amalgams, among which latter he notices the amalgam of silver. He states that it is far superior to gold foil in many particulars, and can be used where the gold foil cannot. Dr. Arthur thinks that this assertion cannot be questioned, but he adds, that the pernicious results observed to follow the use of amalgams very justly discourage its use. The preparation known as sponge gold, crystallized gold, &c., goes far to supply the defects of gold foil. In describing its properties it is stated that sponge gold cannot be plastic, because it is impossible to mould it into a desired form. It simply possesses the property of firmer cohesion, one part with another under pressure; for the purpose of filling, he thinks, this property answers for plasticity. The doctor here goes on to state, from his own experience for several months past, that it takes longer to make a filling with the sponge gold than with the foil, *unless* the operator is content with indifferent operations.

In a large and deep cavity, requiring several layers of gold foil to fill it compactly, and also in very small cavities, the sponge gold may be used to advantage; but in medium sized cavities as good a filling may be made with foil, in less time and with less labor. In regard to the preparation of the cavity in the average number of cases it is necessary to prepare it better for sponge gold. Dr. Arthur prefers the gold made by Watts & Co., possessing a great degree of *adhesiveness* without *friability.*

2.—*Remote Causes of Predisposition to Caries.*—This is the title of a thesis presented to the faculty of the Philadelphia Dental College, by Louis Jack, and published in the News Letter for October.

The author, in the beginning, briefly remarks the wonderful degeneracy of the teeth of the present day, particularly of the rising generation; and thinks that it is a subject worthy the attention of all *inquiring* minds. He shows that man in the primitive condition, when not influenced by the luxuries of civilized life, is seldom subject to the predispositions of disease, and in as far as he is influenced by morbid desires and unnatural customs, so in such proportion will he be subject to physical degeneration and predisposition to disease. Dr. Jack thinks, that predisposition to dental caries may arise, first, from a general interruption of the functions of nutrition during the formation of the teeth. This point is illustrated by noticing the effects marking the teeth of children, who in early life have been subject to disease, especially inflammation of the brain and derangement of the alimentary canal; also by the mother, if cachectic or diseased, during gestation or lactation. Deficiency of the earthy elements in the blood is another point urged. Minute examination of the teeth presents this truth, that in proportion to the relative deficiency of their earthy constituents, are they acted upon by the ordinary exciting causes of caries. The deficiency of phosphate of lime measures their predisposition to decay. Dr. Jack here shows by what circumstances the quantity of lime in the food is influenced; for instance, the soils of the Western States are poorer in lime than the wants of their plants require; they are deficient in phosphates, consequently he thinks that the question naturally arises, "is there not a connection between agriculture and the health of the people?" The early decay of the human teeth has been attributed to the close bolting of flour in its manufacture, as, he says, the greater amount is taken from it in the bran. The quality of the food for the nutrition of the teeth and bones depends upon the amount of phosphates contained in them, and he here goes on to discuss the character of the different kinds of food, the proportion of phosphate contained in them, the circumstances affecting the amount of phosphates, also, the influence of the quality of the metal, and a brief consideration of the differences in solidity between that of the different classes.

---

3.—*Hemorrhagic Diathesis,* is the title of an article in the News Letter, for October, 1854, by Roley Augustine. This article contains some remarks upon the importance of a knowledge of hemorrhagic predisposition to the dentist, and it also points out where an ignorance would lead to serious or fatal results. He say, "as is evident to all in the treatment of disease of any and every nature, the step of paramount importance, is the formation of a correct diagnosis; failing in this, all action is malpractice."

Two cases are given of hemorrhage, in one of which, all other means failing, cobweb was resorted to, saturated in watered borax, rolled in powdered bayberry, and compressed as a plug into the cavity, and it proved successful.

---

4.—*Chloride of Zinc.*—By J. D. White, News Letter, October. This preparation has been used, it is stated by Dr. White, to great advantage in destroying the nervous pulps of teeth. It is made by adding oxyd of zinc to pure muriatic acid by the aid of heat, until no more is dissolved. The solution is evaporated to dryness, rubbed to powder and kept in a closely stoppered vessel. Several cases are related where it was used successfully. In regard to its application, Dr. White says, "we frequently dry the cavity as much as possible, and place a small crystal in contact with the sensitive part, and let it remain until it has deliquesced, when, as a general rule, it will have done its work." In other cases he applies it on a bit of cotton, allows it to remain a day or two and renews it three or four times.

---

5.—*Alloying Gold.*—By S. Duncan, News Letter, October. "Multiply the quantity by its own karat, and divide the product by the karat required; the difference between the quotient and quantity is the necessary alloy,"—"for instance, take a $20 piece, 516 grs.×21.6°=11. 145.6÷18° gives 619.2, from which subtract the quantity, 516, and we have 103.2—the alloy to be added."

---

6.—*Supernumerary Teeth.*—S. V. Howard, News Letter, October, 1854. This gentleman mentions a case in which he extracted a supernumerary tooth on the outer surface of the first molar, upper jaw, left side. It was a small, conical shaped tooth, with a prong nearly three-quarters of an inch long.

7.—*Sponge Gold.*—The January number of the News Letter opens with an article upon Sponge Gold, by J. W. T. Rice, in which he enters into some directions for filling teeth with this preparation of gold, and gives some good advice. This article is followed by remarks by Dr. J. D. White, to the effect that although sponge gold is applicable in some cases, it does not possess advantages over foil in all of them;—the Dr. thinks that nothing is to be gained in economy of time, labor and expense.

8.—*Mineral Plate Bases for Artificial Teeth.*—Mr. Loomis, in the News Letter for January, 1855, says, that the first difficulty to be overcome in the mineral plate process, for sets of teeth, is to make an exact allowance for the shrinkage of the porcelain, in the cast upon which it is moulded, and to do this let the cast, if for an upper half set, be sawed apart in the middle, from front to rear. If the material shrink one-eighth in burning, let those two halves be separated that distance, and the V shaped cavity thus formed be filled with plaster. Then cut the cast crosswise, about one-third of its length from front, the parts separated one-eighth, filled again with plaster. The other part of the matrix the same way. The under cast to be cut crosswise once and finished as before. The difficulties of shrinkage in this way may be *completely* overcome. Mr. Loomis states, that in his experience a plate never warps if properly bedded on the tile, and for this purpose kaolin is recommended. In regard to their splitting or parting, it is advised that they should be heated gradually; they should be cooled, also, slowly. It is thought by the author of this article, that considering the time and labor economised by this process, the day is not far distant when it will be in general use.

9.—*Dental Neuralgia.*—Dr. White, in the January number of the News Letter, relates the case of a young lady, for whom he had filled a right second superior bicuspis, but which, being the cause of severe pain over the whole side of her face, after several efforts to save it, it was extracted. The tooth was cut in two, and the pulp cavity was two-thirds filled with ossific matter, and a serous fluid filling up the space between it and the walls of the dental cavity. Dr. White thinks that the ossific formation was the cause of all the pain.

10.—*Curious Case.*—News Letter, January. Dr. White records the case of a lady, who believed that from some galvanic action upon her food, caused by the use of a plate with three teeth, she diminished in size from 280 pounds to 160; Dr. W. put up another upon purer gold, but as yet there has been no observable increase.

11.—*Difficult Cases of Extraction.*—The Dental Register, for January, 1855, contains an article, with the above heading, by Dr. J. Taylor. The first case related, was that of a first molar of the left side, inferior maxilla; the tooth much decayed and on the palatal face broken down to the alveolus, giving no resting for the inner beak of the forceps. The Dr. thought this could be taken out with the key, but the patient refused—went to another operator, and upon the first application of the forceps the tooth was broken, and with many efforts, after half an hour, the roots were removed. For this case Dr. T. thinks the key invaluable. Several other cases are given in this article, where it was found necessary, in order to extract, to proceed differently than in ordinary cases, illustrating the remark, "that we sometimes encounter teeth which cannot be removed by fair means, but may by foul."

12.—*Gutta Percha Bases for Artificial Teeth.*—N. Y. Dental Recorder, for January, 1855. Dr. Estes, the author of this communication, says:—"After taking the impression and making the metallic dies in the usual way, I take tinned sheet iron and strike up a plate, a little smaller every way than required when done; select and arrange the teeth, try them in the mouth, and then put the whole in plaster and sand, as though for a whole case; remove the wax, line the teeth with the same material as the plate, rivet them, file off and shape the linings with a coarse file so as to leave them rough, return the teeth to their bed in the plaster, wash both the linings and the plate under them with muriate of zinc, and solder by placing small bits of block-tin in the crevices and on the rivets, and melt them with the blow-pipe. The plate and linings already tinned over, the solder will flow finely, holding with greater strength than it otherwise would. After this the plate and linings must be made as rough as possible, the case boiled in saleratus water to remove the muriate of zinc. Then punch as many holes through the plate as possible without springing it; take the male die, oil the surface, and use gutta percha rolled about the thickness of gold plate; soften by heat, press it to the die wherever intended to be covered by the plate. The gutta percha must be softened by dry heat, and the plate of teeth must be placed upon the die covered with the vase before it has time to cool, and held in position until hardened; then, with a hot knife-blade, trim off the superfluous gutta percha, fill all the crevices, and in front imitate the shape of the gum. After this, warm the plate, and another sheet of warm gutta percha must be pressed upon its lingual surface, to touch and unite with the base at the opposite surface of the plate, the points of union being the spots of gutta percha protruding through the small holes. The last layer should be broad enough to be carried over the linings and backings. After this a small piece of gum color is put on, trim the work and it is finished.

